# Alternate typhoid toxin assembly evolved independently in the two *Salmonella* species

**DOI:** 10.1128/mbio.03403-23

**Published:** 2024-03-19

**Authors:** Antonio J. Chemello, Casey C. Fowler

**Affiliations:** 1 Department of Biological Sciences, University of Alberta, Edmonton, Alberta, Canada; University of Washington, Seattle, Washington, USA

**Keywords:** bacterial pathogenesis, pathogen evolution, bacterial evolution, AB_5_-type toxins, typhoid toxin, *Salmonella enterica*, *Salmonella bongori*, typhoid fever

## Abstract

AB_5_-type toxins are a diverse family of protein toxins composed of an enzymatic active (A) subunit and a pentameric delivery (B) subunit. *Salmonella enterica* serovar Typhi’s typhoid toxin features two A subunits, CdtB and PltA, in complex with the B subunit PltB. Recently, it was shown that *S*. Typhi encodes a horizontally acquired B subunit, PltC, that also assembles with PltA/CdtB to produce a second form of typhoid toxin. *S*. Typhi therefore produces two AB_5_ toxins with the same A subunits but distinct B subunits, an evolutionary twist that is unique to typhoid toxin. Here, we show that, remarkably, the *Salmonella bongori* species independently evolved an analogous capacity to produce two typhoid toxins with distinct B subunits. *S. bongori*’s alternate B subunit, PltD, is evolutionarily distant from both PltB and PltC and outcompetes PltB to form the predominant toxin. We show that, surprisingly, *S. bongori* elicits similar levels of CdtB-mediated intoxication as *S*. Typhi during infection of cultured human epithelial cells. This toxicity is exclusively due to the PltB toxin, and strains lacking *pltD* produce increased amounts of PltB toxin and exhibit increased toxicity compared to the wild type, suggesting that the acquisition of the PltD subunit potentially made *S. bongori* less virulent toward humans. Collectively, this study unveils a striking example of convergent evolution that highlights the importance of the poorly understood “two-toxin” paradigm for typhoid toxin biology and, more broadly, illustrates how the flexibility of A-B interactions has fueled the evolutionary diversification and expansion of AB_5_-type toxins.

**IMPORTANCE:**

Typhoid toxin is an important *Salmonella* Typhi virulence factor and an attractive target for therapeutic interventions to combat typhoid fever. The recent discovery of a second version of this toxin has substantial implications for understanding *S*. Typhi pathogenesis and combating typhoid fever. In this study, we discover that a remarkably similar two-toxin paradigm evolved independently in *Salmonella bongori*, which strongly suggests that this is a critical aspect of typhoid toxin biology. We observe significant parallels between how the two toxins assemble and their capacity to intoxicate host cells during infection in *S*. Typhi and *S. bongori*, which provides clues to the biological significance of this unusual toxin arrangement. More broadly, AB5 toxins with diverse activities and mechanisms are essential virulence factors for numerous important bacterial pathogens. This study illustrates the capacity for novel A-B interactions to evolve and thus provides insight into how such a diverse arsenal of toxins might have emerged.

## INTRODUCTION

AB_5_-type toxins are secreted protein complexes composed of an active (A) subunit in complex with a pentameric binding/delivery (B) subunit (1, 2). They are of substantial biomedical importance since they are essential virulence factors for numerous major bacterial pathogens (3–7). AB_5_ toxins enable bacterial pathogens to manipulate host cell biology through the combined activities of their B subunit, which binds specific host cell receptors, triggering toxin uptake and intracellular trafficking, and their A subunit, which enzymatically modifies specific host cell target(s). There is tremendous diversity among AB_5_ toxins with respect to both genetic sequence and biological function (2). The pertussis family of AB_5_ toxins consists of an A subunit that ADP-ribosylates target host cell proteins and a B subunit that binds sialic acid-terminated glycan receptors. The host protein(s) targeted by the A subunit and the sialoglycan-binding specificities of the B subunit vary in different members of this family, which can be found in diverse bacterial lineages including *Bordetella pertussis* (pertussis toxin), *Salmonella* (type 1 and type 2 ArtAB toxins), *Escherichia coli* (EcPlt toxins), and others (2, 8–11). Pertussis family B subunits are particularly diverse and widespread. Outside of conventional pertussis family toxins, they can also be found as components of subtilase toxin, where they form an AB_5_ toxin with an unrelated serine protease A subunit, and as “orphan” B subunits that do not have an overt genetically linked A subunit (12, 13).

**Fig 1:**
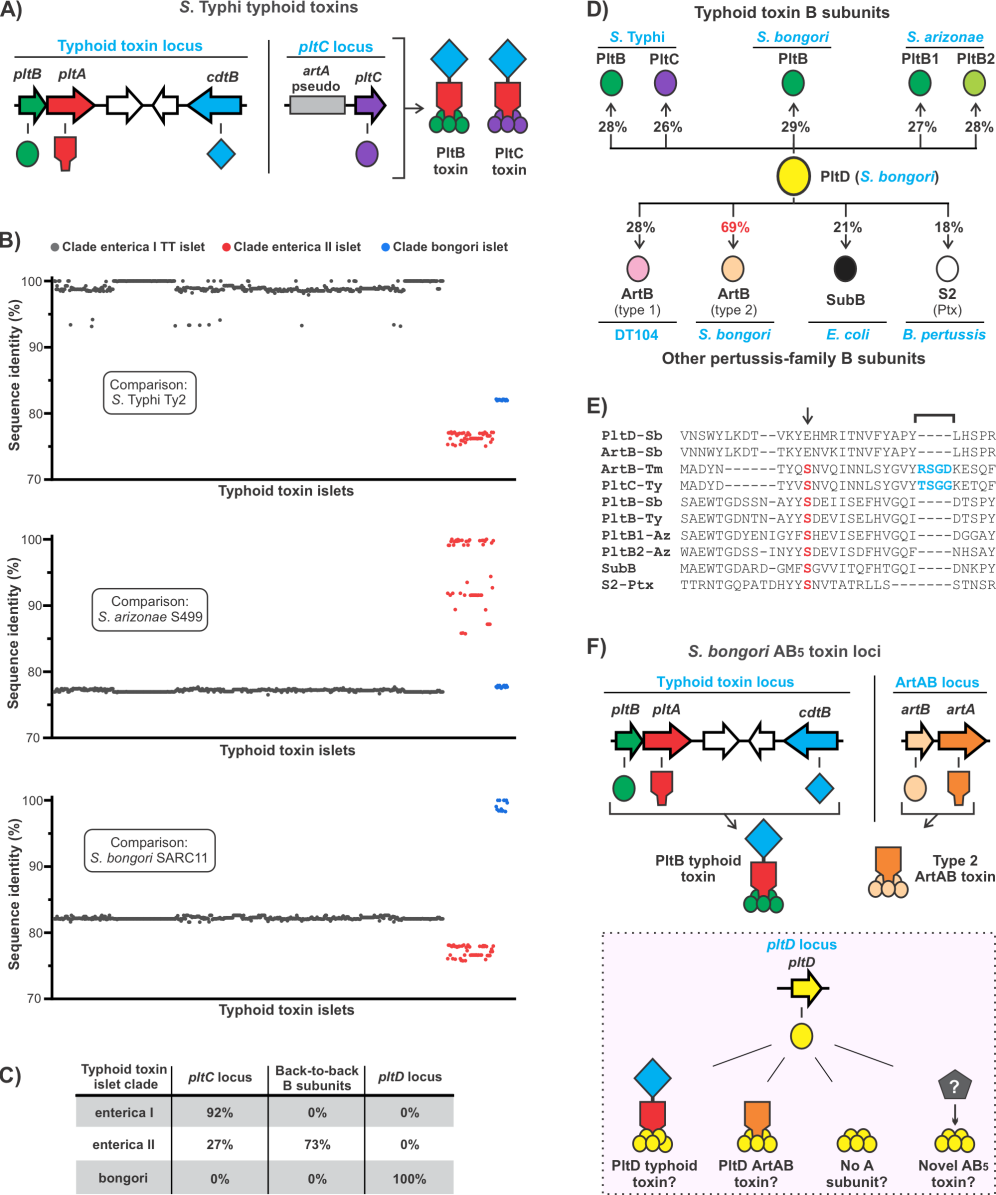
Analysis of the distribution of typhoid toxin genes in *Salmonella* suggests a conserved role for a second typhoid toxin B subunit. (**A**) Schematic representations of the genetic arrangement of typhoid toxin genes and the two typhoid toxins that are produced by *S*. Typhi. (**B**) The percent DNA sequence identity of each typhoid toxin locus identified in the NCBI nr DNA sequence database relative to the representative members of each typhoid toxin clade including *S*. Typhi strain Ty2 (enterica I clade) in the top graph, *Salmonella arizonae* strain S499 (enterica II clade) in the middle graph, and *S. bongori* strain SARC11 (bongori clade) on the bottom graph. The region analyzed spans from the start codon of *cdtB* through the start of the codon of *pltB*. Individual typhoid toxin islets are color coded by clade as indicated. The relationship between sequence diversity and phylogeny within clades is further explored in Fig. S1. (**C**) The percentage of genomes in each typhoid toxin clade that encode the various alleles predicted to serve as a second typhoid toxin B subunit. (**D**) Schematic depicting the percent amino acid sequence identity that PltD shares with diverse pertussis toxin family B subunits. The lineages that encode the various B subunits are in blue text: *S*. Typhi (strain Ty2), *S. bongori* (strain SARC11), *S. arizonae* (strain S499), DT104 (*S*. Typhimurium phage type DT104), *E. coli* (strain 98NK2), and *B. pertussis* (strain Tohama-1). (**E**) Amino acid sequence alignment of the proteins described in panel **D**, showing the region that features a serine residue (arrow, red text) that is essential for receptor binding at the conserved glycan-binding pocket found in diverse pertussis family B subunits, and a short insertion (bracket, blue text) that forms a second glycan-binding pocket in type 1 ArtB and PltC; PltD lacks both of these features. (**F**) Schematic showing the assortment of AB_5_ toxin genes identified in *S. bongori* SARC11, the putative toxins they produce, and four possibilities for the toxin(s) that could be produced using the orphan B subunit PltD. Data relevant for panels B and C can be found in Dataset S1, and data relevant for panels D and E can be found in Fig. S2.

Another toxin that features pertussis family subunits is typhoid toxin, an important virulence factor for the human-adapted pathogen *Salmonella enterica* serovar Typhi (*S*. Typhi), the cause of typhoid fever (4, 14). Typhoid toxin has a pertussis-like AB_5_ core composed of a PltB (B subunit) homopentamer in complex with PltA (A subunit). However, it is distinct from other pertussis family toxins in that it has a unique A_2_B_5_ architecture; PltA forms a disulfide bond with a second A subunit, CdtB, yielding a toxin with two different enzymatic subunits (15, 16) (Fig. 1A). PltA is an active ADP-ribosyltransferase; however, its target(s) have not been identified. All known phenotypes elicited by typhoid toxin are due to CdtB, a DNase that introduces double-stranded breaks into host cell genomic DNA resulting in G2/M cell cycle arrest or cell death (15, 17). Salmonellae are facultative intracellular pathogens that invade host cells and establish a replicative niche known as *Salmonella*-containing vacuole (SCV). Typhoid toxin’s expression is tightly repressed until *S*. Typhi reaches the SCV, at which point high levels of typhoid toxin are produced and then secreted into the SCV (18–20). *S*. Typhi typhoid toxin does not directly intoxicate the cell in which it is produced (17). Instead, PltB binds glycans decorating the mannose-6-phosphate receptor within the SCV membrane, triggering toxin packaging into exocytic vesicles that deliver typhoid toxin to the extracellular space (17, 21, 22). From this location, PltB selectively binds host cell surface receptors featuring *N*-acetylneuraminic acid (Neu5Ac)-terminated sialoglycans, leading to typhoid toxin uptake, intracellular trafficking, and ultimately CdtB-mediated DNA damage (15, 23–25).

The five-gene typhoid toxin islet (Fig. 1A) is exclusively found in *Salmonella*, but it is not restricted to *S*. Typhi (11, 26–28). There are two species within the *Salmonella* genus, *S. bongori* and *S. enterica*, which are predicted to have diverged ~50 million years ago (29). *S. enterica* can be divided into >2,500 serovars, which fall into at least six subspecies. This includes five subspecies (*arizonae*, *diarizonae*, *houtenae*, *indica,* and *salamae*) that primarily inhabit cold-blooded hosts, as well as subsp. *enterica*, which has evolved to infect warm-blooded animals and contains numerous serovars that are important human pathogens such as Typhi, Typhimurium, Enteritidis, and others (30). *S. bongori* is thought to be primarily associated with cold-blooded hosts, and in humans, it appears to only cause disease in young children or immunocompromised individuals (31, 32). It is rarely isolated from environmental or animal sources, but one *S*. *bongori* lineage was endemic to Sicily in the 1980s and 1990s, leading to dozens of clinical cases of acute enteritis in young children (32). Typhoid toxin is found in both *Salmonella* species and assorted subspecies and serovars (11, 26–28). It is evident that it has been subject to substantial horizontal gene transfer, as it has a sporadic phylogenetic distribution and can be found in diverse genome locations (11, 28). Typhoid toxin is thought to contribute to *S*. Typhi’s capacity to establish long-term infections (4). Consistent with this, it is generally absent from serovars that cause short-term infections, but present in salmonellae that establish long-term associations with a host, such as the typhoidal serovars and lineages that asymptomatically colonize cold-blooded hosts (11, 26–28).

It was recently discovered that, surprisingly, *S*. Typhi produces a second version of typhoid toxin that features the same A subunits (PltA and CdtB), but a different B subunit (PltC) (Fig. 1A) (28). PltC and PltB are both pertussis family B subunits, but they share <30% amino acid sequence identity and have different glycan-binding specificities that confer substantial functional differences in cell culture models of infection and in animal models of intoxication (28, 33). *pltC* is encoded immediately downstream of a degraded *artA* gene and this locus exhibits clear sequence homology to loci that encode type 1 ArtAB toxins (11). PltC, therefore, is the result of the evolutionary exaptation of an ArtB subunit that conferred *S*. Typhi with the ability to produce two functionally diverse versions of typhoid toxin (11, 28). This appears to be a widespread feature of typhoid toxin biology since *pltC* loci can be found in diverse typhoid toxin-encoding *Salmonella* lineages, and the Javiana serovar has also been shown to incorporate both PltB and PltC into typhoid toxin (11, 27, 34).

In this study, we analyzed the sequences and distributions of typhoid toxin islets and pertussis family delivery subunits in the *Salmonella* lineage. We determined that, although *pltC* is broadly distributed among *S. enterica* strains that encode typhoid toxin, it is absent from the *S. bongori* species. Remarkably, however, we find that *S. bongori* produces a second version of typhoid toxin in which its A subunits form a toxin complex with a pertussis family B subunit that is evolutionarily distant from both PltB and PltC. We explore the interactions of the *S. bongori* typhoid toxin subunits, as well as the toxicity of the *S. bongori* typhoid toxins against human cells during infection. Collectively, these data reveal that a strikingly similar “two-toxin” paradigm evolved independently in both species of *Salmonella*.

## RESULTS

### Analysis of the distribution of typhoid toxin genes in *Salmonella* identifies PltD, an “orphan” B subunit in *S. bongori*

We set out to examine typhoid toxin genetic diversity and the proportion of typhoid toxin-encoding genomes that feature a second delivery subunit. As a cross-section of *Salmonella* diversity, we analyzed the collection of typhoid toxin islets found in the NCBI nr DNA sequence database, which contains >2,000 complete *Salmonella* genomes from ~200 different serovars. We identified 538 strains that harbor a typhoid toxin islet, ~30% of which were encoded by *S*. Typhi or *S*. Paratyphi A isolates, and the remaining ~70% of which were from isolates scattered throughout the *Salmonella* genus (Dataset S1). On the basis of sequence similarity, the identified typhoid toxin islets clustered into three clades: (i) the “enterica I clade,” which includes the *S*. Typhi islets, the majority of other *S. enterica* subsp. *enterica* islets and a few strains from subsp. *salamae*, (ii) the “enterica II clade,” which appears to be ubiquitous in subsp. *arizonae* and *diarizonae* and can also be found in scattered strains from subsp. *enterica*, *houtenae,* and *salamae*, and (iii) the “bongori clade,” which is found in all 14 *S*. *bongori* genomes in the queried database but is not found in *S. enterica* (Fig. 1B; Dataset S1). There is sequence variation within each clade, with the bongori clade being the most conserved and enterica II clade being the most heterogeneous (Fig. 1B; Fig. S1). However, there is a pronounced separation between the three clades, such that members of a given clade share only 75%–83% DNA sequence identity with the other clades (Fig. 1B); for perspective, the median DNA sequence identity between orthologous *E. coli* and *S. enterica* genes is ~80% (35). Coupled with the broad distribution pattern described above, this suggests that typhoid toxin likely emerged relatively early following the appearance of the *Salmonella* genus and that the three typhoid toxin clades are separated by a substantial evolutionary distance.

We next examined the distribution of auxiliary (in addition to PltB) B subunits within typhoid toxin-encoding genomes. For the enterica I clade, we found that ~92% of genomes encode *pltC*, while most of the remaining ~8% of genomes appear to only encode a single (PltB) B subunit (Fig. 1C; Dataset S1). For the enterica II clade, we found that ~27% of strains encode *pltC*, while the remaining 73% of strains carry two *pltB* homologs back-to-back at their typhoid toxin locus, an arrangement we identified previously (28); we refer to these genes as *pltB1* and *pltB2* (Fig. 1C; Dataset S1). Our analysis thus indicates that the vast majority of *S. enterica* strains that encode typhoid toxin utilize two different typhoid toxin delivery subunits: either PltB1 and PltB2 (generally confined to subsp. *arizonae* and *diarizonae*) or PltB and PltC (the predominant arrangement in other subspecies). Although all of the *S. bongori* strains in the queried database encode typhoid toxin, none encodes *pltC* or back-to-back *pltB* genes, indicating that neither of these arrangements is utilized to diversify the delivery mechanisms of *S. bongori* typhoid toxins (Fig. 1C; Dataset S1). However, upon searching the *S. bongori* genomes for genes related to known AB_5_ toxin B subunits, we identified a gene present in all queried *S. bongori* genomes (SBG_2381 in the SARC11 strain) that shares ~20%–30% amino acid sequence identity with a wide range of pertussis-family AB_5_ toxin B subunits, including PltB and PltC (Fig. 1C and D; Fig. S2). On the basis of the results presented below and in accordance with established typhoid toxin nomenclature, we name this gene *pltD*. The closest relative of PltD is the B subunit of type 2 ArtAB toxins (~70% DNA and amino acid sequence identity), a pertussis-like toxin encoded within prophages found in some *S. bongori* genomes (Fig. 1D; Fig. S2) (11). *pltD* does not share significant DNA sequence similarity with other pertussis-family B subunits, including *pltC* (Fig. S2). Interestingly, an amino acid sequence alignment of assorted pertussis-family B subunits indicates that PltD and type 2 ArtB both lack a serine residue within the conserved glycan-binding pocket that has been shown to be essential for glycan binding in diverse members of this family (Fig. 1E; Fig. S2) (15, 16, 23, 33, 36, 37). PltD and type 2 ArtB also lack the short insertion that forms a second glycan-binding pocket that is unique to PltC and type 1 ArtB (Fig. 1E; Fig. S2) (16, 33). Therefore, PltD does not appear to utilize either of the characterized glycan-binding pockets employed by pertussis B subunits, suggesting it is likely to have unique receptor-binding properties.

*pltD* is found on a small, *S. bongori*-specific islet between the *yfiA* and *pheA* genes (Fig. S2). The genes encoding the A and B subunits of AB_5_ toxins are generally adjacent and co-transcribed; however, there is no gene (or overt pseudogene) immediately upstream or downstream of *pltD*, making *pltD* an “orphan” B subunit. There were, therefore, several possibilities for what PltD’s function might be, including (i) serving as a second typhoid toxin delivery subunit, (ii) serving as a second delivery subunit for the prophage-encoded type 2 ArtAB toxins found in many *S. bongori* genomes, (iii) assembling with a different *S. bongori* enzyme to produce a unique AB_5_ toxin, or (iv) functioning as a B_5_ pentamer only and manipulating host cell biology via interactions with surface receptors, a function that has been proposed for other orphan AB_5_-type B subunits (Fig. 1F) (13). Given the widespread utilization of two typhoid toxin B subunits in *S. enterica*, we hypothesized that PltD might serve as a second typhoid toxin delivery subunit that is unique to the *S. bongori* species.

### The *S. bongori* orphan B subunit PltD is co-expressed with typhoid toxin but not with ArtAB

**Fig 2:**
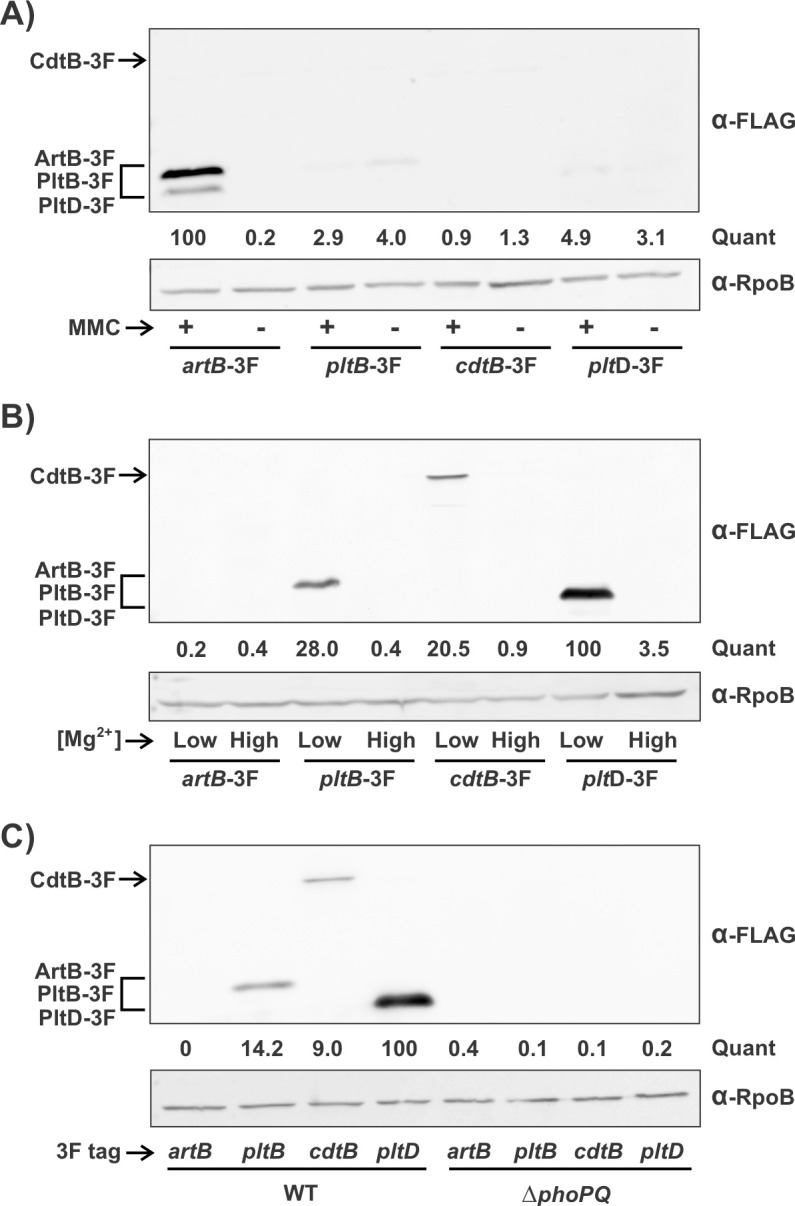
*pltD* and the typhoid toxin genes are co-expressed in a PhoPQ-dependent manner. (**A**) The indicated 3F-tagged *S. bongori* strains were grown in LB or LB containing 0.5 µg/mL MMC for 16 hours, pelleted, and whole cell lysates were analyzed by western blot using an α-FLAG antibody, as well as an α-RpoB antibody, which served as a loading control. (**B**) Similar to panel **A**, except that bacteria were grown in a chemically defined low Mg^2+^ medium, TTIM, or an otherwise identical medium containing high Mg^2+^ levels for 24 hours. (**C**) Similar to **panel B**, except that all samples were grown in TTIM. WT and Δ*phoPQ* strains carrying the four indicated 3F-tagged genes (respectively) were analyzed in parallel to assess the role of PhoPQ in the observed regulation. Quantification of 3F-tagged protein levels (“quant”), performed as described in Materials and Methods, is the average value from at least two independent experiments with similar results. Additional data relevant to this figure can be found in Fig. S3.

We investigated the hypothesis that the orphan B subunit PltD is an alternate subunit for one or both of the AB_5_ toxins encoded by *S. bongori*: typhoid toxin and ArtAB (type 2) toxin. We reasoned that if PltD is a component of one or both toxins, it would need to be co-expressed with the corresponding A subunit(s) under similar environmental conditions. It has previously been reported that *S. bongori*’s ArtAB toxin is induced by the presence of prophage-inducing agents such as mitomycin C (MMC) (38). Although it is not known how typhoid toxin expression is controlled in *S. bongori*, in *S*. Typhi, the typhoid toxin genes are induced via the PhoP/PhoQ (PhoPQ) two-component system in response to conditions encountered within the SCV, such as a low concentration of Mg^2+^ (18). We first generated strains in which C-terminal 3×FLAG (3F) epitope tags were added to ArtB, PltB, CdtB, and PltD, respectively, at their native genomic loci. We then analyzed the levels of these proteins by western blot following growth in LB with and without MMC (Fig. 2A), as well as in a defined growth medium featuring a low concentration of Mg^2+^ (TTIM) or an otherwise identical medium containing high levels of Mg^2+^ (TTIM + Mg^2+^) (Fig. 2B). We found that *S. bongori* produced high levels of ArtB following MMC induction as expected, but that ArtB was undetectable under low Mg^2+^ growth conditions, indicating that the ArtAB toxin is produced under phage-inducing conditions but not under PhoPQ-activating conditions. Conversely, we found that MMC induction did not promote the expression of PltB or CdtB, but that production of both of these typhoid toxin proteins was robustly induced by low Mg^2+^ growth conditions. Importantly, we found that PltD levels tracked with those of the typhoid toxin genes; very high levels of PltD were observed in TTIM, but PltD levels were very low or undetectable in all other conditions (Fig. 2A and B). To confirm these observations, we analyzed transcript levels using RT-qPCR following growth in the same assortment of growth media and found an identical expression pattern; *pltB*, *cdtB,* and *pltD* were all specifically induced in TTIM, whereas *artA* was specifically induced by MMC (Fig. S3). To determine if PhoPQ mediated the observed regulation of the typhoid toxin genes and *pltD* in *S. bongori*, we deleted *phoP*/*phoQ* in each of the 3F strains described above and compared protein levels in the WT and Δ*phoPQ* strains when grown in TTIM (Fig. 2C). We observed that, unlike in the WT strains, PltB, CdtB, and PltD were no longer detectable following growth in TTIM, indicating that PhoPQ is essential for their expression under these conditions. Collectively, these data indicate that PltD is co-expressed with the typhoid toxin genes in a manner that requires PhoPQ and that it is not expressed under conditions that induce the production of the ArtAB toxin.

### PltB and PltD both interact with CdtB in a PltA-dependent manner

**Fig 3:**
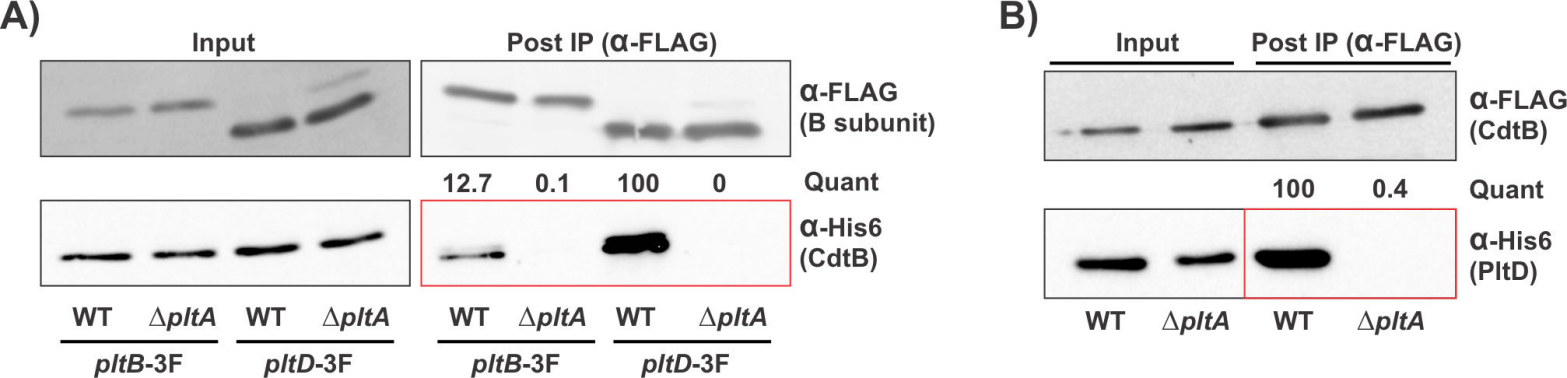
PltD is incorporated into typhoid toxin in *S. bongori*, associating with CdtB in a PltA-dependent manner. (**A**) WT and Δ*pltA cdtB-His_6_* strains, carrying a 3F-tag on either PltD or PltB as indicated, were grown in TTIM for 24 hours. The bacteria were then lysed, and clarified lysates were immunoprecipitated using an α-FLAG antibody. Samples from clarified lysates (input) and elutions from the immunoprecipitation (post-IP) were then analyzed by western blot using both α-FLAG and α-His_6_ antibodies. (**B**) Similar to panel **A**, but using strains in which the epitope tags were switched (*pltD-His_6_*, *cdtB-3F*). Red boxes highlight lanes where interactions are investigated (detecting the protein that lacks the 3F tag targeted by the IP in the elution samples). The average protein levels in post-IP samples (band intensities) were quantified (“quant”) using data from at least two independent experiments with similar results.

Given the co-regulation of PltD with typhoid toxin, we used a co-immunoprecipitation (co-IP) approach to determine if PltD assembles with PltA and CdtB into a toxin complex. For these experiments, we used strains featuring a C-terminal 6×His (His_6_) epitope tag to *cdtB* (to facilitate its detection) that also carried either a *pltB-3F* or *pltD-3F* epitope tag, which was used for IP. We grew these strains in TTIM to induce typhoid toxin expression and immunoprecipitated cell lysates using an α-FLAG antibody. In typhoid holotoxins, the B subunits interact directly with PltA but not with CdtB, whose incorporation into the toxin is contingent on its association with PltA (Fig. 1A). We, therefore, generated Δ*pltA* versions of these strains and tested them in parallel as controls for the co-IP experiment. Western blot analysis of the IP samples from these experiments indicated that CdtB-His_6_ was detected in the immunoprecipitation fractions for both PltB and PltD, with greater amounts of CdtB in the PltD sample, suggesting that both PltB and PltD form a complex with CdtB (Fig. 3A). Importantly, CdtB-His_6_ did not co-IP with PltB or PltD in strains featuring a *pltA* deletion, despite being present at equivalent levels in the input fractions. This indicates that the interaction of both PltB and PltD with CdtB is dependent on PltA, consistent with the established typhoid toxin architecture. To verify these observations, we generated strains in which the epitope tags were reversed (*cdtB-3F + pltD-His_6_*, WT, and ΔpltA) and conducted analogous co-IP experiments. Mirroring the previous results, PltD-His_6_ co-immunoprecipitated with CdtB-3F when PltA was present but not in the Δ*pltA* strain (Fig. 3B). These results therefore indicate that *S. bongori* produces typhoid toxins that incorporate both PltB and PltD as binding subunits.

### PltB and PltD do not interact but compete for active subunits to form distinct typhoid toxins

**Fig 4:**
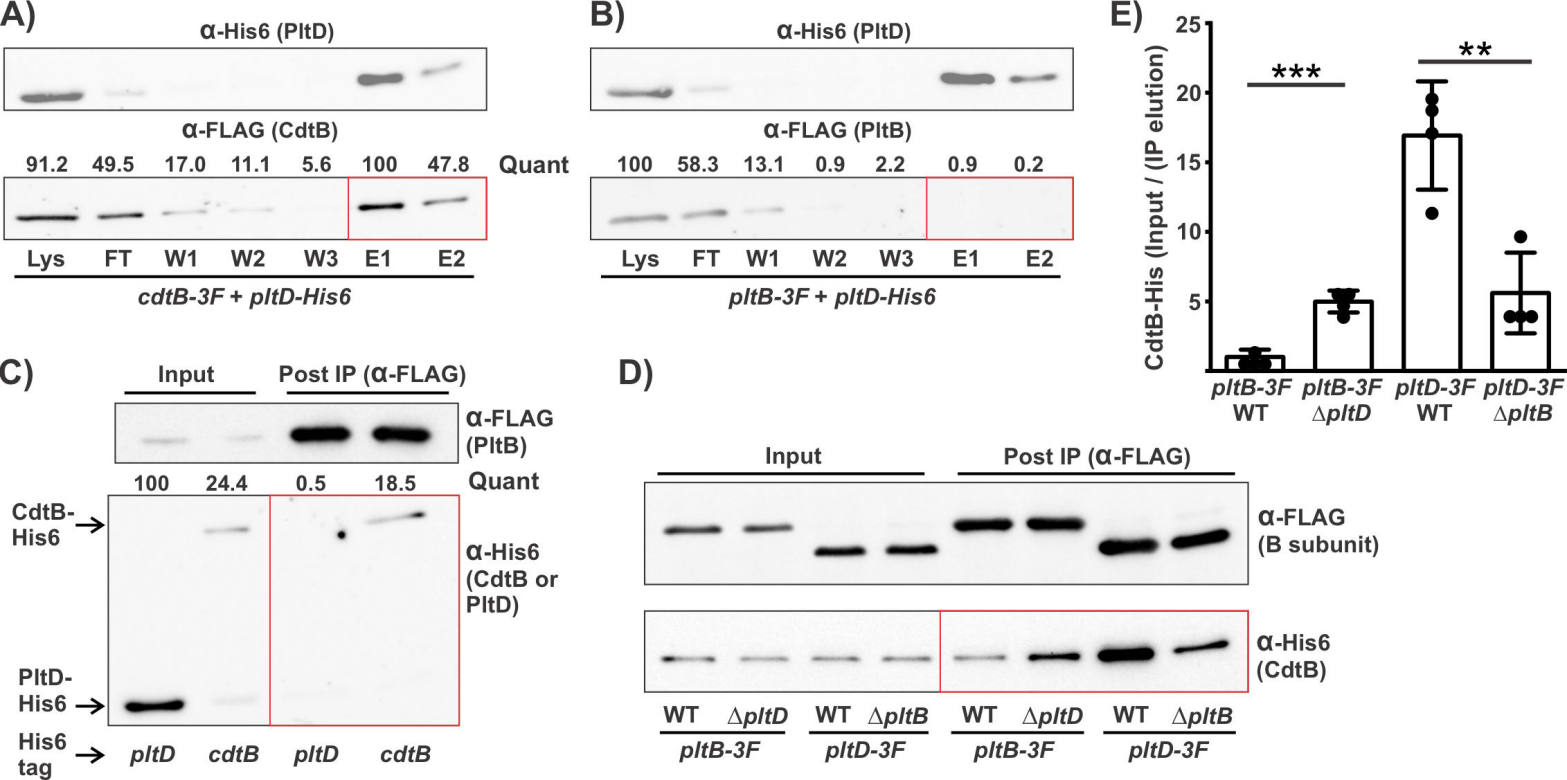
PltD and PltB do not interact, but compete for A subunits to form distinct typhoid holotoxins. (**A**) and (**B**) *pltD-His_6_* strains carrying either *cdtB-3F* (**A**) or *pltB-3F* (**B**) were grown for 24 hours in TTIM and then lysed. PltD-His was then purified from clarified lysates using immobilized metal affinity chromatography using Nickel-NTA columns. Western blot analysis was used to detect the levels of both PltD-His and the indicated 3F-tagged protein in the clarified lysate (Lys), the flowthrough (FT), the washes (**W1–W3**), and the elutions fractions (**E1 and E2**). (**C**) *pltB-3F* strains, carrying a His_6_-tag on either PltD or CdtB as indicated, were grown in TTIM for 24 hours. Bacteria were then lysed, and clarified lysates were immunoprecipitated using an α-FLAG antibody. Samples from clarified lysates (input) and elutions from the immunoprecipitation (post-IP) were then analyzed by western blot using both α-FLAG and α-His_6_ antibodies. Average band intensities from at least two independent experiments were quantified (“quant”) for the 3F-tagged proteins in panels A/B, and for His_6_-tagged proteins in panel C, as described in Materials and Methods. (**D**) Similar to panel **C**, but using a different assortment of strains. All strains carried a *cdtB-His_6_* tag and a 3F tag on either *pltB* or *pltD*, as indicated. For each of these strains, WT strains and strains in which the untagged B subunit was deleted were tested in parallel to examine the effect of deleting one B subunit on the toxin levels for the other B subunit. (**E**) Quantification of the ratio of the abundance of CdtB-His_6_ in the elution samples compared to the levels in the input (clarified lysate) from the experiment described in panel **D**. Bars represent the average ratios from four independent experiments, and error bars represent standard deviations. For each 3F-tagged B subunit, values for the WT strain and the strain with a deletion to the other B subunit were statistically compared using two-tailed *t*-tests: **P < 0.01 and ***P < 0.001. Red boxes highlight lanes where interactions are investigated (detecting the protein that lacked the epitope tag used for purification/immunoprecipitation in the elution samples).

Although AB_5_ toxins generally employ a single, homopentameric B subunit, pertussis toxin and *S*. Typhi typhoid toxin are exceptions to this and utilize multiple different B subunits. However, they do so in a fundamentally different manner: pertussis toxin assembles as a single toxin that utilizes a heteropentameric delivery platform composed of four different B subunits that serve different structural/functional roles within the complex, whereas *S*. Typhi typhoid toxin is deployed as two separate toxins that feature different homopentameric delivery platforms that confer distinct functional characteristics (28, 39). We therefore examined whether *S. bongori* produces a single typhoid toxin with a PltB/PltD heteropentameric delivery platform or two separate typhoid toxins featuring PltB and PltD B subunits, respectively. To assess this, we conducted immobilized metal affinity chromatography (IMAC) using Nickel-NTA columns to selectively purify PltD-His_6_ and, by association, any proteins that form stable interactions with PltD (Fig. 4A and B). We performed this experiment using *pltD-His_6_* strains that feature 3F tags on either CdtB or PltB to enable us to detect interaction (co-purification) with these proteins. We observed that, for both strains, PltD-His_6_ was abundant in the IMAC elution fractions, indicating that it was efficiently purified using this method. Consistent with the results above, CdtB-3F was also readily detected in the elution, indicating that it interacts with PltD (Fig. 4A). By contrast, although PltB-3F was readily detectable in the input fractions, it was undetectable in the elution fractions, suggesting that PltD does not interact with PltB (Fig. 4B). To confirm this result, we conducted co-IP experiments using an α-FLAG antibody to immunoprecipitate PltB-3F in strains carrying either CdtB-His_6_ or PltD-His_6_. Western blot analysis indicated that, although PltD-His_6_ produced a much stronger band than CdtB-His_6_ in the input samples, CdtB-His_6_ was found in the IP samples, whereas PltD-His_6_ was not (Fig. 4C). Together, these results indicate that PltB and PltD do not form heteropentameric delivery platforms, but rather that (like *S*. Typhi) *S. bongori* produces two different typhoid toxins featuring distinct PltD and PltB homopentameric delivery platforms.

In *S*. Typhi, the PltB and PltC B subunits compete for A subunits, with PltC exhibiting the capacity to outcompete PltB (28, 33). As a result, deletion of *pltC* causes increased production of the PltB typhoid toxin both *in vitro* and during infection (28). To explore B subunit competition in *S. bongori*, we compared the levels of CdtB-His_6_ that co-immunoprecipitated with each of the B subunits in a wild-type background compared to strains where the other B subunit was deleted (Fig. 4D). In a WT background, we observed that substantially greater amounts of CdtB immunoprecipitated with PltD-3F compared to with PltB-3F (an average of >15-fold stronger band intensity by western blot analysis), suggesting that the PltD toxin is the more abundant of the toxins when *S. bongori* are grown in TTIM (Fig. 4D and E). Consistent with this, we found that substantially more (approximately fivefold) CdtB immunoprecipitated with PltB-3F in the Δ*pltD* strain compared to WT, indicating that greater levels of PltB typhoid toxin are produced when PltD is absent from the cell, suggesting the B subunits compete for A subunits (Fig. 4D and E). Conversely, deletion of *pltB* did not increase the amount of CdtB in the PltD-3F co-IPs; in fact, there was a reduction in the amount of CdtB in the IP of the Δ*pltB* strain (Fig. 4D). The Δ*pltB* mutation did not reduce *pltA* transcript levels (in fact, we observed a very minor increase, Fig. S4); we speculate this effect might be due to impacts on PltA translation, folding, or stability (see Discussion). Collectively, these data suggest that PltB and PltD compete for A subunits and that, like in *S*. Typhi, the “alternate” B subunit appears to be able to outcompete PltB.

### Deletion of *pltD* leads to increased intoxication of cultured human cells during infection

**Fig 5:**
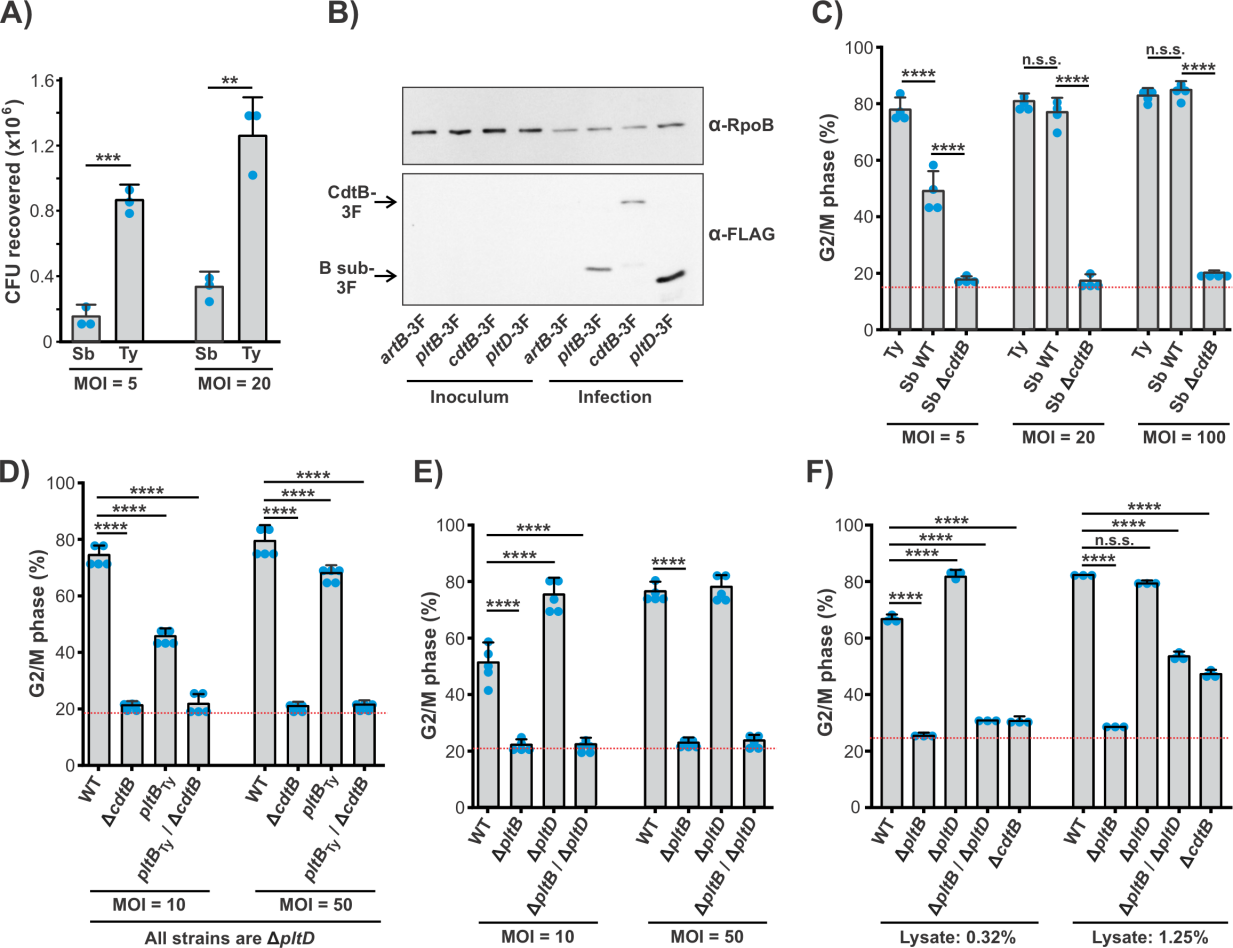
Intoxication of human cells by *S. bongori* typhoid toxins. (**A**) WT *S. bongori* (Sb) and WT *S*. Typhi (Ty) were used to infect HeLa cells at the indicated MOIs. Gentamycin was added to the growth medium to prevent the growth of extracellular bacteria. Cells were collected, and bacteria were isolated 48 hpi and plated on LB-agar plates to determine the number of CFU recovered. Statistical significance was determined by two-tailed *t*-tests: **P < 0.01 and ***P < 0.001. Error bars represent one standard deviation. (**B**) *S. bongori* strains encoding the indicated 3F-tagged genes were used to infect HeLa cells at an MOI of 50. Bacteria were extracted at 24 hpi, pelleted, and whole cell lysates were analyzed by western blot using α-FLAG and α-RpoB (loading control) antibodies. A second replicate of this experiment is shown in Fig. S8. (**C**) *S*. Typhi (Ty), *S. bongori* WT (Sb WT), and *S. bongori* Δ*cdtB* (Sb Δ*cdtB*) strains were used to infect HeLa cells. At 72 hpi, cells were collected and fixed using ethanol. Cell cycle analysis was performed on PI-stained cells as described in Materials and Methods to determine the levels of CdtB-induced G2/M cell cycle arrest. Bars represent the average percentage of cells in the G2/M phase for four biological replicates. (**D and E**) The indicated *S. bongori* strains were used to infect HeLa cells, and those cells were incubated, collected, and analyzed as in panel C. Bars represent the average percentage of cells at the G2/M phase from five biological replicates. All strains in panel D have Δ*pltD* mutations; *pltB*_Ty_ strains encode the *S*. Typhi *pltB* gene in place of the native (*S. bongori*) *pltB* gene. (**F**) Clarified lysates (containing soluble material from both host and bacterial cells) from HeLa cells infected with the indicated *S. bongori* strains were used to treat (uninfected) HeLa cells for 72 hours with the indicated amount of lysate. Cells were collected and analyzed as in panel C. Bars represent the average percentage of cells in the G2/M phase for three biological replicates from a single experiment; four additional similar experiments were conducted and yielded comparable results. For panels C–F, the red dotted lines indicated the average %G2-M phase of uninfected control cells, error bars represent one standard deviation, and statistical significance of the indicated comparisons was determined by Tukey’s test: n.s.s., not statistically significant and ****P < 0.0001. Additional data relevant for panels C–F can be found in Fig. S3; data relevant for panel D can be found in Fig. S4.

We next examined the activity of *S. bongori*’s typhoid toxins using a cell culture model of infection. Infections of cultured human epithelial cell lines have been used extensively as a model system for investigating the activity and biological program of *S*. Typhi typhoid toxin. Consistent with a previous study, we found that *S. bongori* is able to establish an infection of cultured HeLa cells (31). At multiplicities of infection (MOIs) of 5 and 20, substantial numbers of *S. bongori* were recovered at 48 hours post-infection (hpi) and only approximately three- to sixfold fewer bacteria than were recovered from *S*. Typhi infections at the same MOIs (Fig. 5A). This indicates that *S. bongori* is able to establish an infection in this cell line and that it persists within these cells for an extended period of time. Western blot analysis of lysates from infected cells indicated that expression of 3F-tagged versions of CdtB, PltB, and PltD were all induced upon infection, whereas ArtB-3F expression was undetectable (Fig. 5B). This is congruent with the expression analyses above and indicates that, like *S*. Typhi, *S. bongori* specifically expresses its typhoid toxin genes during infection.

To examine the activity of the *S. bongori* typhoid toxins, we performed cell cycle analysis on infected cells. Because CdtB elicits DNA damage that culminates in G2/M cell cycle arrest, analyzing the proportion of cells at the G2/M phase of the cell cycle is a well-established means of tracking typhoid toxin intoxication. As expected, infection with a WT *S*. Typhi strain (positive control) resulted in very high levels of G2/M cell cycle arrest, whereas infection with a Δ*cdtB S. bongori* strain (negative control) exhibited similar proportions of cells at the G2-/M phase as uninfected controls (Fig. 5C). Surprisingly, we found that infection with WT *S. bongori* led to very high proportions of intoxicated cells, even at modest MOIs. Indeed, although somewhat higher levels of intoxication were observed for *S*. Typhi compared to *S. bongori* at low MOIs (Fig. 5C), given that *S*. Typhi infections yield higher bacterial loads (Fig. 5A), *S. bongori* appears to intoxicate this cell line with a similar efficiency as *S*. Typhi on a per-bacterium basis. To explore this issue further, in the *S. bongori* Δ*pltD* genetic background, we generated strains in which the *pltB* from *S*. Typhi was inserted into the genome in place of the native *pltB* gene (*pltB*_Ty_). Because the B subunit is often observed to drive functional differences between different AB_5_ toxin sequence variants or subtypes, we reasoned that this would allow us to evaluate whether the *S*. Typhi *pltB* had evolved to more efficiently target human cells compared to the *S. bongori pltB*. To test PltB_Ty_ production levels and toxin incorporation, we also generated a 3F-tagged version of the *pltB*_Ty_ strain and found that the *pltB*_Ty_ strain produced somewhat reduced levels of PltB-3F compared to wild-type strains that encode the native *pltB* (Fig. S6). However, we found that the levels of CdtB-His_6_ that co-IP with strains that encode WT *pltB* or *pltB*_Ty_ were indistinguishable, indicating that PltB_Ty_ efficiently assembles into typhoid holotoxin (Fig. S6). We then infected cells using the *pltB*_Ty_ and WT strains, as well as Δ*cdtB* controls, and examined typhoid toxin activity via cell cycle analysis (Fig. 5D). All of these strains also featured a Δ*pltD* mutation to eliminate the variables of PltD activity or competition. We found that both the WT and the *pltB*_Ty_ strain efficiently intoxicated cells, with the *pltB*_Ty_ strain exhibiting modestly reduced intoxication compared to WT. These results are not consistent with the premise that *S*. Typhi PltB is substantially more efficient at targeting human cells than *S. bongori* PltB. Rather, these data suggest that, in the context of this specific cell culture model of infection/intoxication, *S. bongori* PltB is surprisingly efficient at mediating typhoid toxin delivery.

We next examined the contributions of the different *S. bongori* B subunits to its typhoid toxin activity during infection. Deletions to the various *S. bongori* typhoid toxin subunits did not significantly impact the number of CFU recovered from infection (Fig. S7). As expected, we found that strains featuring a deletion of both B subunits failed to intoxicate cells, even at a high MOI (Fig. 5E). Interestingly, we found that a Δ*pltB* strain also failed to intoxicate cells, indicating that the PltB typhoid toxin is solely responsible for the intoxication observed in this infection model. Consistent with this, infection with the Δ*pltD* strain elicited significantly greater levels of intoxication than the WT strain at an MOI of 10 (intoxication is at the apparent maximum of ~80% for the Δ*pltD* strain at MOI = 10; the WT strain catches up to this level at MOI = 50). Coupled with the results described above (Fig. 4D and E), these data suggest that deletion of *pltD* leads to increased levels of PltB typhoid toxin and thus increased typhoid toxin activity. We reasoned that the PltD typhoid toxin (i.e., Δ*pltB* strains) might fail to intoxicate cells in this infection model because it is unable to engage with exocytic machinery and thus remains trapped in the SCV. To explore this possibility, we developed an intoxication assay in which cell lysates from *S. bongori*-infected cells were added to fresh (uninfected) cells. This material does not include toxins secreted into the cell culture medium (exocytosed toxin) but instead represents a crude mixture of the human and bacterial lysates from infected cells, including cell-associated toxins. As expected, due to the high levels of various bacterial and host cell products, this lysate is toxic to cells in a typhoid toxin-independent manner when added at high concentrations. More dilute samples, however, were well tolerated by cells and found to elicit G2/M cell cycle arrest in a CdtB-dependent manner (Fig. 5F), indicating that this experiment could measure the activity of the pool of typhoid toxin associated with infected cells. Cells given lysate from Δ*pltB*/Δ*pltD* infections showed indistinguishable proportions of cells at the G2/M phase compared to the Δ*cdtB* control, indicating that B subunit-mediated toxin delivery was still required in this assay and that the crude lysate did not promote spurious CdtB cell uptake. As observed for the infection assays, the Δ*pltD* strain was more potent than the WT strain in the lysate intoxication assay, whereas the Δ*pltB* strain was completely inactive (Fig. 5F). These results suggest that the lack of typhoid toxin activity observed for infections with the Δ*pltB* strain is not strictly due to inefficient PltD-mediated toxin exocytosis, but rather that the PltD typhoid toxin is ineffective at intoxicating human epithelial cells. Collectively, these data suggest that the evolutionary acquisition of the PltD subunit reduced the potency with which *S. bongori* intoxicates human cells during infection.

## DISCUSSION

It was recently discovered that *S*. Typhi produces two different versions of typhoid toxin that have the same A subunits but different B subunits (28). This appears to have evolved as a result of the exaptation of a B subunit from a distantly related pertussis family toxin to serve instead as an alternate typhoid toxin B subunit. This evolutionarily acquired B subunit, PltC, is expressed from a different locus but is able to outcompete PltB for A subunits and forms the dominant toxin under conditions tested to date (28, 33). The PltC toxin is not efficiently exported from the SCV and thus strains lacking *pltB* exhibit greatly reduced toxicity upon infection of cultured mammalian cells (28). Conversely, strains lacking *pltC* produce greater levels of PltB typhoid toxin and more potently intoxicate human cells during infection than WT strains do. In this study, we show that a strikingly similar situation evolved independently in the other *Salmonella* species, *S. bongori*. Like PltC, PltD is expressed from a locus that is distant from the other typhoid toxin genes, but it is able to outcompete PltB for inclusion in typhoid toxin and is the dominant toxin produced under the conditions tested. Also, similar to the situation with PltC, strains producing only PltD typhoid toxin fail to intoxicate host cells during infection, whereas strains lacking *pltD* produce increased PltB typhoid toxin and elicit increased CdtB-mediated toxicity compared to wild-type strains during infection of human cells. We provide genetic evidence that the “two-toxin” paradigm is nearly ubiquitous among typhoid toxin-encoding *S. enterica* strains (Fig. 1C). Coupled with the discovery that this capacity evolved independently in *S. bongori*, this strongly suggests that the two-toxin paradigm is a fundamental and important aspect of typhoid toxin biology.

There are several possible evolutionary benefits of producing two similar toxins with different receptor-binding subunits. One possibility is that this diversifies the cell/tissue types that can be intoxicated during infection. Indeed, the only other known AB_5_ toxin with multiple different B subunits, pertussis toxin, binds cells in a promiscuous manner due to the numerous distinct glycan-binding sites on its S2 and S3 B subunits (37, 40, 41). This is thought to be a driving force behind the diverse phenotypes that can be associated with this toxin during *B. pertussis* infection (2, 7, 8). Another possibility is that the benefit of producing two different toxins relates to the unique intracellular production of typhoid toxin and the associated need for B subunit-driven exocytosis of the toxin from the SCV to the extracellular space prior to toxin activity. Interestingly, in *S*. Typhi, the PltC toxin remains in the SCV in cell culture models of infection (28), which suggests that the different versions of the toxin might be deployed differently. There might be a benefit to maintaining a pool of toxins within the SCV that would then be released in the event of host cell lysis, since toxins freed from the lysed cell could potentially protect the released bacteria from detection by the host immune system. Alternatively, this second pool of toxins might be exported only by specific host cell types or only when specific conditions in the host cell are encountered. Selective deployment of the two toxins would not work with a single heteropentameric toxin (like pertussis toxin) and would therefore explain why PltC and PltD have not evolved to form heteromeric delivery complexes with PltB. The alternative deployment hypothesis also explains why the two-toxin paradigm has seemingly not evolved in any of the other (many) species that produce AB_5_ toxins, since all other known AB_5_ toxins are produced by extracellular bacteria and their trafficking does not involve this exocytic stage.

There are several questions that emerge from this study. Although we have shown that PltD is a *bona fide* typhoid toxin subunit, it is not impossible that it also interacts with additional proteins. PltD’s glycan-binding properties are an interesting area for future investigation. Sequence analysis suggests that PltD lacks conserved elements of the established glycan-binding pockets in related B subunits, and our functional data suggest that PltD toxins might not efficiently engage HeLa cell receptors. Given the natural host range of *S. bongori*, it is possible that the glycans bound by PltD might not be present or abundant in mammals. Questions also remain concerning the competition of the two B subunits for assembling with PltA. *pltB* and *pltA* are separated by ~15 bp in both *S. bongori* and *S*. Typhi and are known to be co-transcribed in *S*. Typhi (18). It is therefore likely that their translation and Sec-mediated secretion into the periplasm are coupled as well, which would seemingly provide PltB with the upper hand in its competition with PltD. In this context, it is surprising that PltD appears to be the dominant toxin. For *S*. Typhi, it has been shown that, *in vitro*, PltC is able to displace PltB from its holotoxin to form a toxin with PltA/CdtB, whereas PltB is not able to displace PltC from its holotoxin (33). Given that the folding and assembly of A and B subunits are thought to be coordinated for certain AB_5_ toxins (42), one model for toxin assembly would be that co-translated PltA and PltB assemble into a toxin first and that PltC (in *S*. Typhi) or PltD (in *S. bongori*) is able to subsequently displace PltB. If true, this would raise the intriguing question of how, mechanistically, one B subunit displaces another. In this study, we observed reduced amounts of PltD typhoid toxin in Δ*pltB* strains compared to WT (Fig. 4D and E) that was not due to reduced *pltA* transcription. The simplest explanation for this is that the *pltB* deletion—in which the first five and last five codons of *pltB* were left intact to avoid polar effects—reduced the efficiency of *pltA* translation or that PltA is misfolded and/or degraded at an increased rate in the absence of co-translated PltB. Future experiments will be required to dissect the mechanisms salmonellae utilize to assemble two different toxins and the effects of factors such as the timing and levels of expression, as well as genetic localization (co-translation) on B subunit competition for PltA.

In many instances, the properties of different sequence variants or subtypes of AB_5_ toxins are known to vary considerably, and these functional differences most often track to the B subunit (2). Indeed, the *S*. Javiana typhoid toxin exhibits substantially reduced toxicity compared to *S*. Typhi typhoid toxin in both cell culture and animal models of intoxication due to three amino acid differences in PltB (43). In this context, and given that *S. bongori* is not thought to have evolved to interact with a mammalian host, we were surprised to observe that *S*. Typhi and *S. bongori* elicited similar levels of G2/M cell cycle arrest upon infection of cultured HeLa (human) cells. *S. bongori*’s surprising toxicity is due to its PltB typhoid toxin since a strain that produces only the PltD toxin did not show any signs of CdtB-mediated toxicity, even at a high MOI. In fact, even lysates from infections using strains that only produce the PltD toxin failed to induce toxicity, suggesting that this is not merely due to an inability to trigger exocytosis. Furthermore, a strain lacking *pltD*, which produces increased amounts of PltB toxin, elicited significantly greater toxicity than WT *S. bongori*. Further studies will be required to determine whether the *S. bongori* PltB typhoid toxin is highly potent against diverse mammalian cell and tissue types, or whether this is an anomaly of the particular cell type used in this study. It is noteworthy that many of the *S*. Typhi PltB residues that are important for its interaction with sialoglycan receptors (e.g., Y33, Y34, S35, K59, T65, R100, and V103) are conserved in *S. bongori* (Fig. S2) (23). Regardless, *S. bongori* lacks the SPI-2 locus that is required for efficient growth and survival of salmonellae in mammalian macrophages and is clearly not well adapted to infect a healthy adult human host (44–46). Therefore, despite the key role typhoid toxin is proposed to play in *S*. Typhi pathogenesis, it is unlikely that typhoid toxin would enable *S. bongori* to cause a typhoid fever-like disease in a mammalian host, and such a phenomenon has not been reported in a human patient to our knowledge. However, in instances where this organism gains a foothold in a human host, its typhoid toxin’s potent activity against epithelial cells could contribute to human disease, such as by eliciting tissue damage that promotes intestinal inflammation. In this context, the evolutionary acquisition of *pltD*, which appears to have blunted CdtB-mediated toxicity against human cells, might have led *S. bongori* to be less virulent within a human host.

## MATERIALS AND METHODS

### *In silico* analysis of typhoid toxin and B subunit sequences and distributions

To identify the complete set of *Salmonella* genomes within the NCBI nr DNA sequence database that encode a typhoid toxin islet, we used iterative BLASTn searches of this database. The query sequence we used as a starting point was the *S*. Typhi Ty2 typhoid toxin islet sequence spanning from the start of the *cdtB*-coding region through the start of the *pltB*-coding region, capturing the sequence of all five genes. The resulting list of hits was filtered to remove any sequences that were not from complete *Salmonella* genomes, as well as any hits that aligned over <60% of the query sequence. We then identified the hit with the lowest percent sequence identity and used its complete typhoid toxin islet sequence as the query for a second BLASTn search, the results of which were amalgamated with the first search. We continued this process a total of four times, each time identifying the typhoid toxin islet that was the least similar to any islets queried to date and then using this as the next query sequence. Hits that aligned over less than 93% of the query sequence were investigated manually, and we discarded any islets that did not encode intact *cdtB*, *pltA,* and *pltB* genes or that contained a transposon insertion; 12 hits were discarded on this basis. Importantly, all BLASTn searches identified an identical set of genomes that encode typhoid toxin islets, suggesting that the thresholds we set effectively captured islets with diverse sequences. The three clades of typhoid toxin islets were identified on the basis of overt natural break points in the percent sequence identity data when analyzing the iterative BLASTn searches; they are defined here on the basis that each member of a given clade has ≥10% greater sequence identity to the representative member of its own clade than to the representative member of any other clade. Determining which genomes encode a *pltC* locus was done as described previously (11) using BLASTn searches of the *S*. Typhi *pltC* locus (encompassing *sty1362* and *pltC*) and filtering to remove any hits that did not continuously align over >90% of the query sequence. To identify any genomes with back-to-back typhoid toxin B subunits, the *S*. Typhi PltB and PltC amino acid sequences were used in tBLASTn searches (word size = 2, no filtering for low complexity regions), and genomes with two significant hits (*E* value < 0.01) that had genome coordinates that were within 2,000 bp were predicted to have back-to-back B subunits. To confirm this, individual genomes were investigated manually, which confirmed the presence of two distinct, adjacent B subunit homologs encoded on the same strand at the typhoid toxin locus of all genomes identified to have back-to-back B subunits. Typhoid toxin-encoding genomes that lacked both *pltC* and back-to-back B subunits were investigated for the presence of additional B subunits using tBLASTn searches using as query sequences: *S*. Typhi PltB, *S*. Typhi PltC, *S. bongori* PltB, *S. arizonae* PltB2, and *E. coli* SubB, which led to the identification of PltD. To determine which genomes encode PltD, a similar tBLASTn search was conducted, and the results were cross-referenced with the complete list of genomes that harbor typhoid toxin islets. The genomic regions of all hits (>80% query coverage and >50% sequence identity) were investigated manually for the presence of an *artA* gene to differentiate between *pltD* loci and type 2 *artBA* loci. *pltD* and *artBA* loci were found to be easily distinguishable on the basis of sequence: all PltD hits were found to be >97% identical to the PltD query sequence, whereas all type 2 ArtB hits were <71% identical. DNA sequence comparisons of the *pltD* locus compared to the *S. bongori artBA* locus and the *S*. Typhi *pltC* locus were conducted using BLASTn analysis (“align two sequences” setting). Multiple sequence alignments of pertussis-family B subunits were conducted using the EMBL-EBI suite of alignment tools using Clustal Omega (default parameters; Gonnet transition matrix, 6-bit gap opening penalty, 1-bit gap extension penalty). These alignments were used to generate a phylogenetic tree using the Molecular Evolutionary Genetics Analysis V11 software with the parameters noted in the figure legend.

### Bacterial strains, human cell lines, and culture conditions

*S. bongori* strains were derived from the wild-type strain SARC11, also known as *S. bongori*, strain 12419, which was originally isolated from an African frog in 1972 (31, 47). Mutant strains were constructed using standard recombinant DNA and allelic exchange procedures using pSB890 as the vehicle for genetic transfer and *E. coli* β-2163 ∆*nic35* as the conjugative donor strain (48, 49). For strains featuring 3×FLAG tags, tags were introduced to the C-terminus of the indicated gene in all instances and at the native genomic locus unless otherwise indicated. The amino acid sequence of the 3×FLAG epitope tag was DYKDHDGDYKDHDIDYKDDDDK. Deletions of specific genes generally involved the clean removal of the entire coding sequence (start codon to stop codon) without introducing a scar. For *pltB* deletions, because *pltA* resides ~15-bp downstream and altering its expression/translation would impact downstream experiments, we maintained the first five and last five amino acids of the *pltB*-coding sequence (removing all intervening sequences). Bacteria were cultured in either LB broth (routine growth) or in TTIM (to induce toxin expression). TTIM is a chemically defined medium based on N minimal medium (50), modified to optimize typhoid toxin gene expression in *S*. Typhi (18). TTIM (low [Mg^2+^]) contained 10 µM Mg^2+^, whereas TTIM + Mg^2+^, the equivalent high [Mg^2+^] medium, contained 10 mM Mg^2+^. Where indicated, mitomycin C (Sigma) was added to the medium at a concentration of 5 µg/mL.

Cell culture experiments were performed using the human epithelial cell line HeLa (CCL-2), which were cultured in Dulbecco’s modified Eagle medium (DMEM, Gibco) supplemented with 10% fetal bovine serum in a 5% CO_2_ humidified incubator at 37°C. We routinely tested this cell line for mycoplasma contamination using the Myco-Sniff Mycoplasma PCR Kit (MP Biomedicals).

### RT-qPCR analysis of transcript levels

Strains were grown as indicated, after which RNA was isolated using either (i) the RNeasy RNA isolation kit (Qiagen) following supplier instructions for Gram-negative bacteria or (ii) using a hot acid phenol RNA extraction method based on a previously described method (51). Independent experiments using these two different extraction methods yielded similar results. For hot acid phenol RNA extractions, briefly, 2–5 mL of culture was pelleted and resuspended in TES (10 mM Tris HCl pH 7–8, 10 mM EDTA, and 0.5% SDS). Two sequential extractions were then performed using acid phenol:chloroform (Ambion), followed by one chloroform extraction, and then RNA precipitation using isopropanol. Pellets were washed twice with 70% ethanol and resuspended in RNase-free water. RNA was treated with RNase-free DNase I (Invitrogen) and purified by phenol-chloroform extraction followed by ethanol precipitation. RNA extracted by either method was then analyzed by taking OD 260/230 and OD 260/280 measurements using a Nanodrop (Thermo). cDNA was synthesized from purified RNA using Superscript II (Thermo Fisher) according to the manufacturer’s protocol for random hexamer priming. The qPCR was performed using SYBR green supermix and the 7500 Fast Real Time PCR machine (Applied Biosystems) using a 40-cycle, two-step PCR program, followed by a melting curve, which was analyzed to ensure that a uniform PCR product was generated. Transcript levels were determined using primers specific for the indicated genes, and serially diluted *S. bongori* genomic DNA was used to generate standard curves for each primer set to facilitate the standardization of the data. The transcript levels for test genes were normalized to the constitutively expressed control gene, *dsbC*.

### Western blot analysis

Samples were electrophoresed using 10%–14% SDS-PAGE gels and transferred to nitrocellulose membranes (Bio-Rad). Membranes were blocked using 5% non-fat milk and then incubated overnight at 4°C with the indicated primary antibody. The primary antibodies (animal, dilution used, source) used here were monoclonal α-FLAG M2 (mouse, 1:5,000, Sigma), monoclonal α-RpoB (mouse, 1:5,000, BioLegend), and polyclonal α-His-6 (rabbit, 1:20,000, Invitrogen). After repeated washes, membranes were then incubated with a secondary antibody for 2 hours at room temperature covered from light using a 1:15,000 dilution of either IRDye 680RD Goat anti-Mouse IgG (for FLAG/RpoB blots) or IRDye 800CW Goat anti-Rabbit IgG (for His6 blots), which were sourced from Li-Cor, Inc. After repeated washes, membranes were visualized with a Bio-Rad ChemiDoc imaging system. To prepare samples for western blot analysis in experiments that analyzed protein levels in whole cell lysates under various growth conditions, strains were grown as indicated, after which the OD_600_ was measured for normalization. A volume of 1.4 mL of each culture was pelleted and then resuspended in a volume of 2× SDS buffer that normalized for differences in cell number (OD_600_).

Band quantification was performed using the ImageJ image processing and analysis software (https://imagej.nih.gov/ij/), where two methodological approaches were used. In Fig. 2 and 4 (panels A–C), the average band intensities of each lane from independent experiments were determined, removing average background intensity and using the sliding paraboloid background subtraction tool. The highest intensity was normalized to 100. In Fig. 3 and 4D and E , a different method was used to account for the strength of signals in the input samples. The average band intensity of the input (lysate) samples was used to normalize band intensities from independent experiments. Two sample *t*-tests were used to determine the statistical significance of the differences observed in protein levels when comparing the indicated samples.

### Immunoprecipitation experiments

The indicated strains were grown in 28 mL of TTIM for 24 hours, pelleted, and then resuspended in lysis buffer (50 mM Tris pH 7.5, 167 mM NaCl, 1 mg/mL lysozyme, and 1 mM PMSF) and lysed using an EmulsiFlex-B15 High Pressure Homogenizer (Avestin). The lysate was clarified by centrifugation at 20,000 *g* for 15 minutes at 4°C, and a small aliquot was mixed with SDS-loading buffer and set aside (pre-IP “input” sample). Clarified lysates were then incubated with ANTI-FLAG M2 Affinity Gel (Sigma) at 4°C overnight. The M2 affinity gel (agarose beads) was washed four times using lysis buffer, followed by a final wash in lysis buffer with 0.1 M galactose. Immunoprecipitated proteins were then eluted by incubating the M2 agarose beads with SDS-PAGE loading buffer (lacking β-mercaptoethanol and bromothymol blue) at 98°C and 900 rpm in an orbital shaking heat block for 3.5 minutes. After separating the supernatant (elution) from the beads, β-mercaptoethanol and bromothymol blue were added to the elution. Input (pre-IP) and elution (post-IP) samples were then analyzed by western blot as described above.

### Immobilized metal affinity chromatography

Cultures were grown and lysed as in immunoprecipitation experiments. Soluble fractions were added to Ni-NTA spin columns (NEB) and washed and eluted according to the manufacturer’s instructions. Briefly, soluble fractions were incubated in the column for 2 minutes prior to the first spin. Columns were then washed three times with wash buffer containing 20 mM imidazole and finally eluted twice with elution buffer containing 500 mM imidazole. Input samples (clarified lysates) and elution samples were then analyzed via western blot as described above.

### HeLa cell infections

The indicated *S. bongori* or *S*. Typhi strains were grown overnight in LB and then diluted 1/20 in 0.3 NaCl LB media and grown to an OD_600_ of 1.05, at which point the bacteria were added to HeLa cells in HBSS (Gibco) according to the specified MOI. After a 1-hour incubation, cells were washed twice with HBSS to remove extracellular bacteria and incubated for 1 hour in a medium containing 100 µg/mL gentamicin. Cells were then incubated in media containing 5 µg/mL gentamicin for the duration of the infection. To determine the number of CFU, cells were released from the plates using trypsin, washed with PBS, and then lysed by resuspending in 0.1% sodium deoxycholate. Bacterial cells were pelleted at 5,000 × *g* for 8 minutes and resuspended in PBS. CFU plating was performed and, if applicable, the remaining resuspension was pelleted and resuspended in 2× SDS page for western blotting following the protocol previously outlined. For western blot analysis of *S. bongori* protein expression during infection, three 10 cm plates, with 5 × 10^6^ HeLa cells each, were infected. For experiments determining the number of CFU for different strains/time points, 6-well plates with 2 × 10^5^ HeLa cells were used.

### Cell cycle intoxication assays

Quantification of CdtB-mediated cellular intoxication of host cells was done using propidium iodide (PI) staining followed by flow cytometry-based cell cycle analysis, as previously described (15, 28). For infection-intoxication experiments, 2 × 10^5^ HeLa cells were infected as described above using the indicated strains/MOI in 6-well plates. At 72 hpi, cells were collected, washed, and resuspended in 300 µL of PBS. Cells were then fixed while vortexing at low speed by adding 700 µL of ice-cold 100% ethanol in a dropwise fashion, and samples were then stored at −20°C for at least 24 hours. To stain the fixed cells, cells were washed once with 500 µL of PBS and stained with 400 µL of PI dye solution [50 µg/mL PI (Sigma), 0.1 µg/mL RNase A, 5 mM EDTA, and 0.05% Triton X-100 in PBS] for 30 minutes at 37°C. Stained cells were analyzed via flow cytometry using the gating strategy outlined in Fig. S6 using either a Fortessa X-20 or an Attune NxT flow cytometer. To evaluate PI fluorescence, an excitation of 561 nm and an emission of 610/20 or 620/20 nm were used. Results were analyzed using FlowJo (Treestar), and the proportion of gated cells with a DNA content indicative of a G2-M stage of the cell cycle was determined.

For intoxication experiments using cell lysates from infected HeLa cells, 5 × 10^6^ HeLa cells were infected in 10 cm dishes at an MOI of 50. At 72 hpi, infected cells were detached, pelleted, and resuspended in 5 mL of DMEM containing 100 µg/mL gentamicin. Infected HeLa cells were then lysed using an EmulsiFlex-B15 High Pressure Homogenizer (Avestin). Crude lysates were clarified by centrifugation at 5,000 × *g* for 8 minutes, after which the soluble fraction was passed through a 0.2 µm filter to remove any remaining intact human or bacterial cells. The indicated percentages of filtered and clarified infection lysates were then added to 2 × 10^5^ HeLa cells in 6-well plates and incubated for 72 hours, at which point cells were collected and analyzed for intoxication as described above.

## SUPPLEMENTAL MATERIAL

Data Set S1 mbio.03403-23-s0001.xlsx Summary of data used to analyze typhoid toxin islet types and the additional B subunits found in the same genome.

Supplemental Table and Figures mbio.03403-23-s0002.pdf Table S1 and Fig. S1 to S8.

ASM does not own the copyrights to Supplemental Material that may be linked to, or accessed through, an article. The authors have granted ASM a non-exclusive, world-wide license to publish the Supplemental Material files. Please contact the corresponding author directly for reuse.
