# Supplementary material for: Alternate typhoid toxin assembly evolved independently in the two Salmonella species
Source: mBio. 2024 Mar 19;15(4):e03403-23. doi: 10.1128/mbio.03403-23 (PMC11005416; doi:10.1128/mbio.03403-23)
Supplement: Supplemental Table and Figures — Table S1 and Fig. S1 to S8. [file mbio.03403-23-s0002.pdf]

Supplementary Table 1: List of bacterial strains used in this study

**Strain**	**Relevant genotype**	**Reference**
SARC 11	Wild type *S. bongori*	PMID: 8975610, PMID: 21876672
CCF0060	SARC 11: *artB*-3x FLAG	This study
CCF0040	SARC 11: *cdtB*-3x FLAG	This study
CCF0041	SARC 11: *pltB*-3x FLAG	This study
CCF0042	SARC 11: *pltD*-3x FLAG	This study
CCF0062	SARC 11: *artB*-3x FLAG, Δ*phoPQ*	This study
CCF0049	SARC 11: *pltB*-3x FLAG, Δ*phoPQ*	This study
CCF0046	SARC 11: *cdtB*-3x FLAG, Δ*phoPQ*	This study
CCF0047	SARC 11: *pltD*-3x FLAG, Δ*phoPQ*	This study
CCF0048	SARC 11: *pltB*-3x FLAG, *cdtB*-His6	This study
CCF0063	SARC 11: *pltB*-3x FLAG, *cdtB*-His6, Δ*pltA*	This study
CCF0044	SARC 11: *pltD*-3x FLAG, *cdtB*-His6	This study
CCF0064	SARC 11: *pltD*-3x FLAG, *cdtB*-His6, Δ*pltA*	This study
CCF0108	SARC 11: *pltD*-His6, *cdtB*-3x FLAG	This study
CCF0112	SARC 11: *pltD*-His6, *cdtB*-3x FLAG, Δ*pltA*	This study
CCF0107	SARC 11: *pltD*-His6, *pltB*-3x FLAG	This study
CCF0104	SARC 11: Δ*pltD, pltB*-3x FLAG, *cdtB*-His6	This study
CCF0102	SARC 11: Δ*pltB, pltD*-3x FLAG, *cdtB*-His6	This study
CCF0164	SARC 11: Δ*cdtB*	This study
CCF0069	SARC 11: Δ*pltB*	This study
CCF0103	SARC 11: Δ*pltD*	This study
CCF0170	SARC 11: Δ*pltB,* Δ*pltD*	This study
CCF0172	SARC 11: Δ*pltD,* Δ*cdtB*	This study
CCF0167	SARC 11: Δ*pltD, S.* Typhi *pltB*	This study
CCF0173	SARC 11: Δ*pltD, S.* Typhi *pltB,* Δ*cdtB*	This study
CCF0174	SARC 11: Δ*pltD, S.* Typhi *pltB*-3x FLAG, *cdtB*-His6	This study
ISP2825	Wild type *S.* Typhi	PMID: 1879916

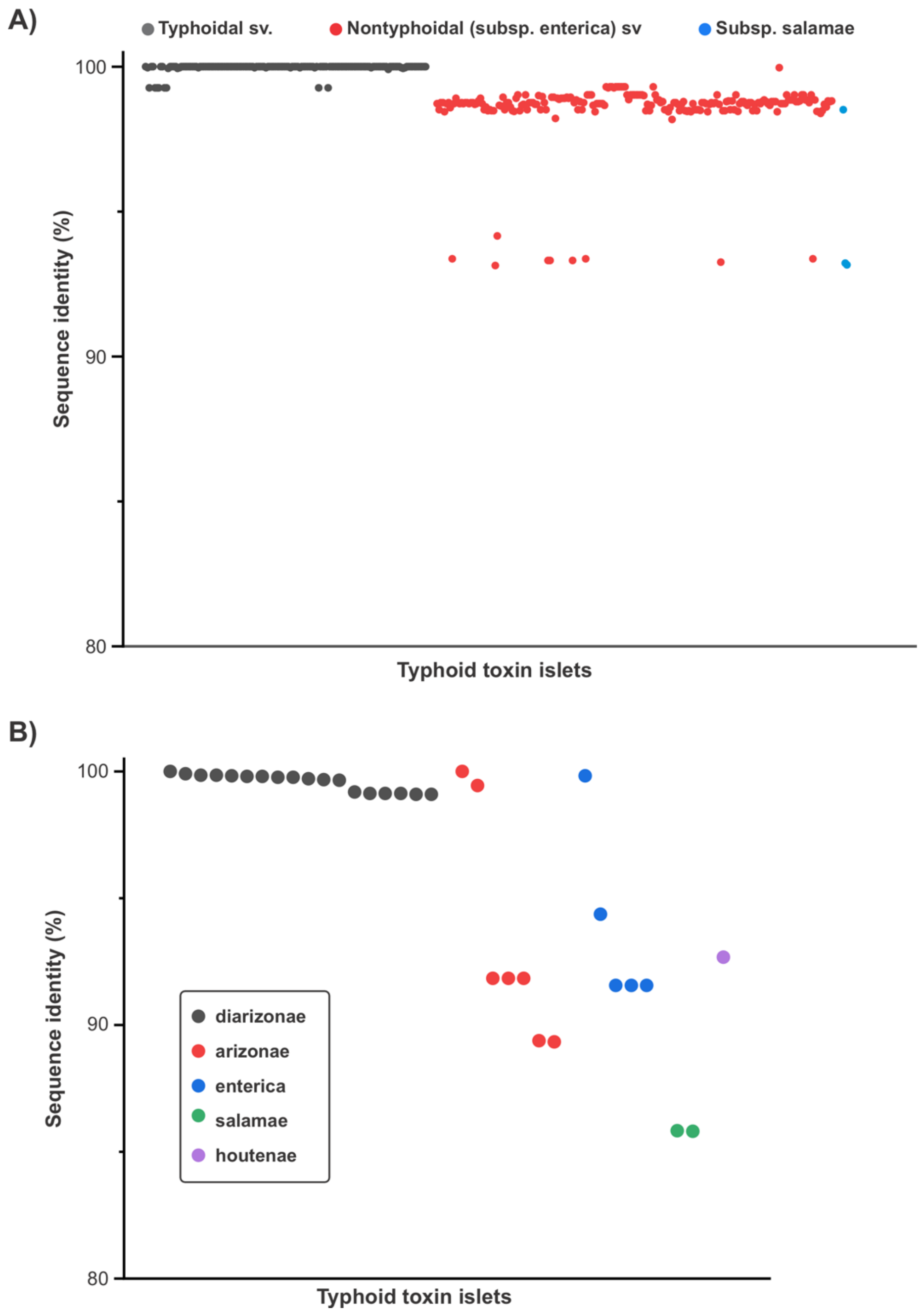

Fig. S1: Sequence diversity within typhoid toxin islet clades as a function of lineage. The percent DNA sequence identity of typhoid toxin islets for the enterica I clade **(A)** or the enterica II clade **(B)** relative to the representative members of that clade, broken down by relevant phylogenetic groups. Clade enterica I (panel A) comparisons are to *S*. Typhi strain Ty2 and are colour coded by typhoidal strains (sv. Typhi and Paratyphi A), nontyphoidal subsp. enterica strains, and subsp. salamae strains as indicated. Clade enterica II comparisons (panel B) are to *S. arizonae* strain S499 and are colour coded by subspecies as indicated. These analyses use the same raw data as the analyses in Fig 1C and are intended to highlight phylogenetic relationships within clades. For both panels, the specific lineage of numerous database sequences was not available and thus these sequences were excluded from this analysis. Raw data for these analyses can be found in Supplemental Dataset 1.

A)

```
PltD-Sb     MMKKKIFSICAFLSLIGSYDSNAAVNSWYLKDT--VKYEHMRITNVFYAPY----LHSPR  54
ArtB-Sb     --MKKIFFAFALVMMAGASNVYATVNNWYLKDT--TKYENVKITNVFYAPY----LHSPR  52
ArtB-DT104  --MKNKLKVLALTLASLSSVCYANMADYN------TYQSNVQINNLSYGVYRSGDKESQF  52
PltC-Ty     --MKKKLKVLTLALASISSVCYAAMADYD------TYVSNVQINNLSYGVYTSGGKETQF  52
PltB-Sb     --MNKIVPVCTLLSLAC---SFYASAEWTGDSSN-AYYSDEIISEFHVGQI----DTSPY  50
PltB-Ty     MYMSKYVPVYTLLILIY---SFNASAEWTGDNTN-AYYSDEVISELHVGQI----DTSPY  52
PltB1-Az    --MKKITSVCALLSLIC---SFNASAEWTGDYENIGYFSHEVISEFHVGQI----DGGAY  51
PltB2-Az    --MKKIFSVCALLITAW---PLSSWAEWTGDSS-INYYSDEVISDFHVGQF----NHSAY  50
SubB        MTIKRFFVCAGIMGCLS---LNPAMAEWTGDARD-GMFSGVVITQFHTGQI----DNKPY  52
S2-Ptx      ------------------------TTRNTGQPATDHYYSNVTATRLLS-------STNSR  29

PltD-Sb     ICAFFTAQPGK--QNVTGCAVADNSYYAKNNAGTSPFDEIFNTLRYFYATGEDISVYIRS  112
ArtB-Sb     ICAYFTASSGG--SNVTGCAVADNGYYQKNAGQTSPFMEIFDTVKYFYTTGEKISVYIRI  110
ArtB-DT104  FCVGLKRGSQV-PNVHTICKIDVFGTH------KQGFDNMLATARYYYATGEDVRIYYKE  105
PltC-Ty     FCIGLKHGSEA-ISINAMCKVDVYGNH------KQGFDNMLNTAKYYYTTGGDVRIYYKE  105
PltB-Sb     FCIKAVKANGS-GTPVVACSVAKQSIW------APSFKELLDQARYFYSTGQAVRIHVQQ  103
PltB-Ty     FCIKTVKANGS-GTPVVACAVSKQSIW------APSFKELLDQARYFYSTGQSVRIHVQK  105
PltB1-Az    FCIKAVKADGSRSTPLIACSVSNESVW------APSFKVLLEQARYFYVTEQSVRIYYDH  105
PltB2-Az    FCIKTVKKSGE-GTPIIACALSHDSKW------IPSFNIMLEQARNFYITGHSIRVYVQP  103
SubB        FCIEGKQSA---GSSISACSMKNSSVW------GASFSTLYNQALYFYTTGQPVRIYYKP  103
S2-Ptx      LCAVFVRSG---QPVIGACTSPYDGKY------WSMYSRLRKMLYLIYVAGISVRVHVSK  80

PltD-Sb     NAF--SDFDTSISRNEIVAIGTCNG--WCFGETIR----------  143
ArtB-Sb     NAF--SHFDSSVSQNEIVAIGTCNQ--WCFGEIIK----------  141
ArtB-DT104  NVWTDRNFTAAFSGNELIAITTCTSSDYCMGPTLPN---------  141
PltC-Ty     NVWRDPDFKSAFSSRELIAITTCSSSSYCMGPTVTN---------  141
PltB-Sb     NIWTNQDFINTFSTNALVGLSSCSA-TQCFGPQ------------  135
PltB-Ty     NIWTYPLFVNTFSANALVGLSSCSA-TQCFGPK------------  137
PltB1-Az    NVWTNQPFVNTFSTNALVGLSSCSAATDCFGPGKPKSESKRDVEQ  150
PltB2-Az    NVWSNKSFIEALSSNALVGLSTCST-SECFGPVKPKM--------  139
SubB        GVWTYPPFVKALTSNALVGLSTCTTSTECFGPDRKKNS-------  141
S2-Ptx      EEQYYDYEDATFETYALTGISICNPGSSLC---------------  110
```

B)

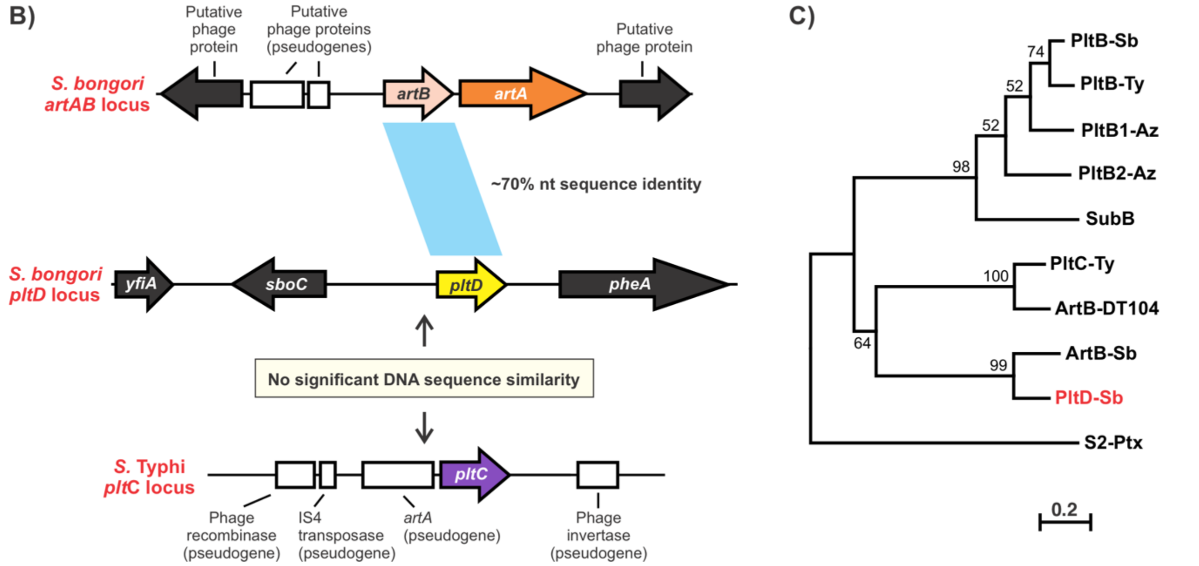

Fig. S2: Phylogenetic and sequence analysis of PltD. **(A)** Amino acid sequence alignment comparing the sequence of PltD to diverse pertussis toxin family B subunits. Sb, *S. bongori* strain SARC11; Ty, *S*. Typhi strain Ty2; Az, *S. arizonae* strain S499; DT104, *S*. Typhimurium phage type DT104; SubB sequence is from *E. coli* strain 98NK2 and S2 (pertussis toxin) sequence is from *B. pertussis* strain Tohama-1. Alignment generated using Clustal Omega. A snippet of this alignment is shown as Fig 1E. **(B)** Genome diagram showing the regions of significant DNA sequence similarity between the *S. bongori pltD* locus and the *S. bongori artBA* locus (top) or the *S*. Typhi *pltC* locus (bottom). **(C)** Phylogenetic tree showing predicted evolutionary relationships for the pertussis family B subunits described in (A). The tree was generated with the MEGA (Molecular Evolutionary Genetics Analysis) V11 software using the maximum likelihood method and a WAG +G +I substitution model. A bootstrap method with 500 total replicates was used, and the numbers at the nodes represent the support values.

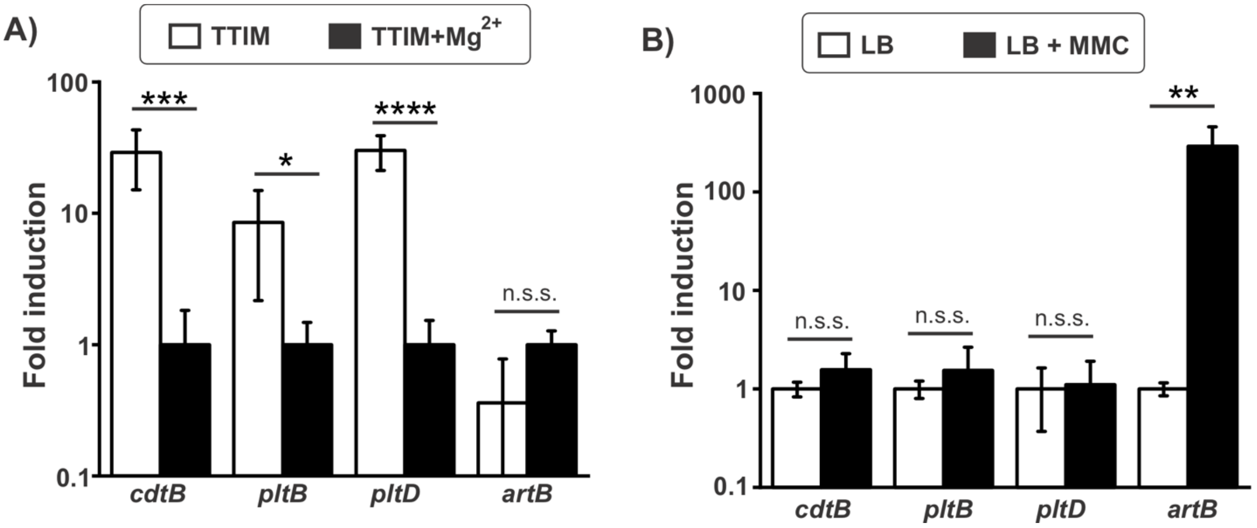

Fig. S3: RT-qPCR analysis of *S. bongori* toxin gene expression indicates that *pltD* is co-regulated with typhoid toxin genes. WT *S. bongori* (SARC 11) was grown in either TTIM and TTIM+Mg for 24 hours **(A)**, or LB with and without 0.5 μg/ml MMC for 16 hours **(B)**, at which point RNA was isolated and transcript levels were analysed using RT-qPCR. Transcript levels of the indicated genes were normalized to a constitutively expressed control gene (*dsbC*). Bars represent the average fold increase in RNA levels for the indicated genes over at least five independent samples from two separate experiments in the inducing condition [TTIM in (A), +MMC in (B)] compared to the non-inducing condition [TTIM+Mg^{2+} in (A), LB only in (B)], and error bars represent the standard deviation. Two-tailed t-tests were used to determine the significance of the induction observed for each gene under each growth condition: * = $p < 0.05$, ** = $p < 0.01$, *** = p , 0.001, **** = $p < 0.0001$, n.s.s. = not statistically significant.

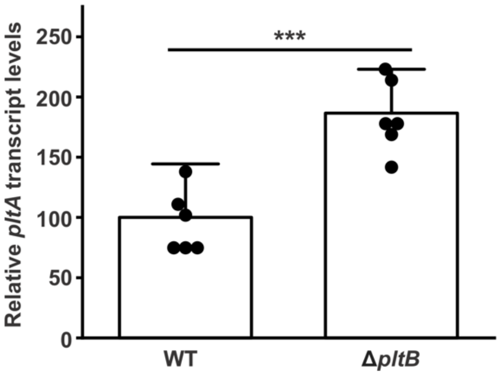

Fig S4: RT-qPCR analysis of the effect of the Δ*pltB* mutation on *pltA* transcript levels. WT and Δ*pltB* *S. bongori* strains were grown TTIM for 6 hours at which point RNA was isolated and transcript levels were analysed using RT-qPCR. *pltA* transcript levels were normalized to a constitutively expressed control gene (*dsbC*). Bars represent the average relative transcript levels for *pltA* over three biological replicates from two separate experiments (n=6). Two-tailed t-tests were used to investigate the statistical significance of any differences in *pltA l*evels observed between the WT and Δ*pltB* strains. *** = p, 0.001.

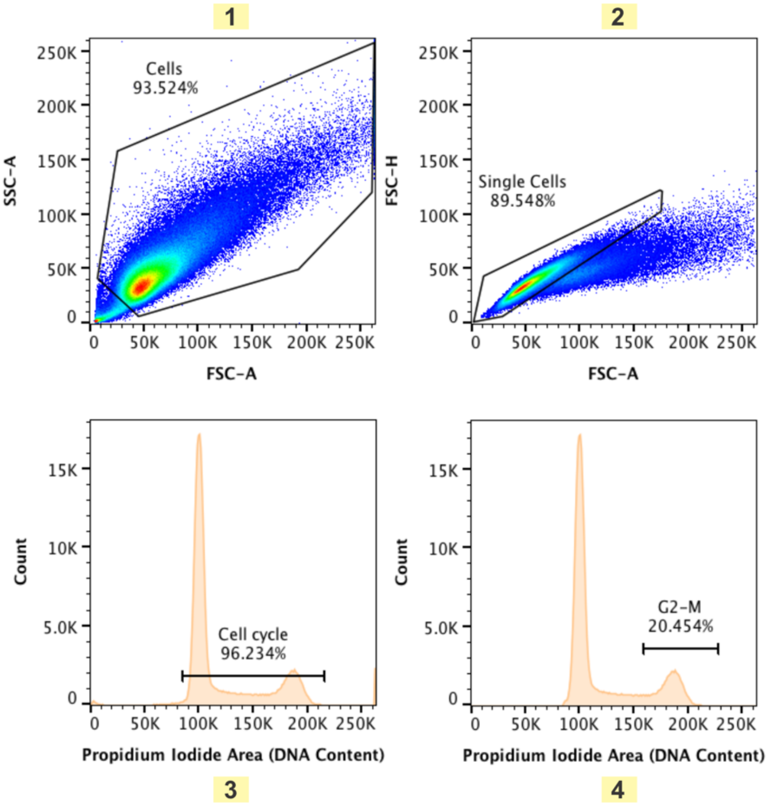

Fig. S5: Example of the gating strategy used for analysis of flow cytometry data for cellular intoxication assays. Gating to analyze cell cycle using PI-stained cells was accomplished using a four-step strategy, as shown above. Particles were first gated for size using FSC area / SSC area to omit cellular debris (1), followed by selection for single cells (doublet omission) using FSC height / FSC area (2), followed by gating on fluorescence intensity in the channel used to detect PI fluorescence in order to omit any remaining particles with a fluorescence intensity outside the minimum (G1 population) and maximum (G2-M population) expected of single cells (3), followed by the final step of identifying the G2-M peak used for intoxication analysis (4). Results shown are from a typical unintoxicated/uninfected control sample. Note that somewhat larger particles were included in our gating to account for CdtB-mediated cellular distension, which is known to yield cells with an atypically large volume.

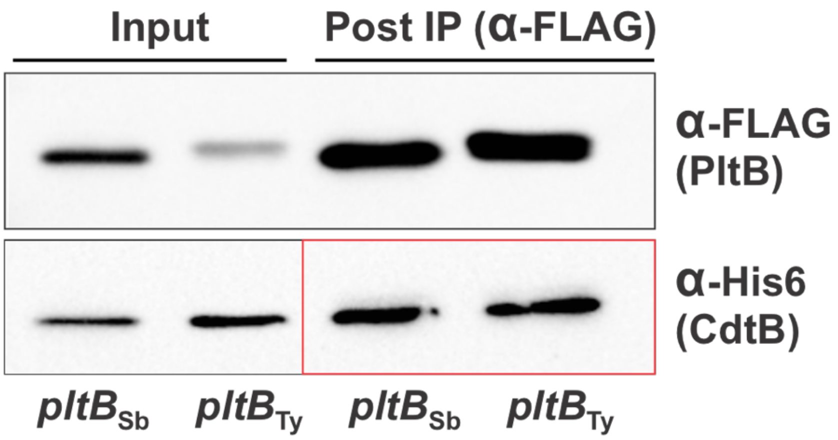

Fig. S6: Protein levels and toxin formation for *S*. Typhi PltB encoded in place of the native *S. bongori pltB*. Two *cdtB-His*$_6$ *S. bongori* strains, *pltB-3F* (*pltB*$_{Sb}$) as well as a strain in which the native *pltB* gene was replaced with *S*. Typhi *pltB-3F* (*pltB*$_{Ty}$), were grown in TTIM for 24 hours. The bacteria were then lysed, and clarified lysates were immunoprecipitated using an α-FLAG antibody. Samples from clarified lysates (input) and elutions from the immunoprecipitation (Post-IP) were then analyzed by western blot using both α-FLAG and α-His6 antibodies. The red box highlights the lanes where interactions are investigated (detecting the protein in the elution samples that lacks the 3F tag targeted by the IP). This experiment was conducted independently two times with equivalent results.

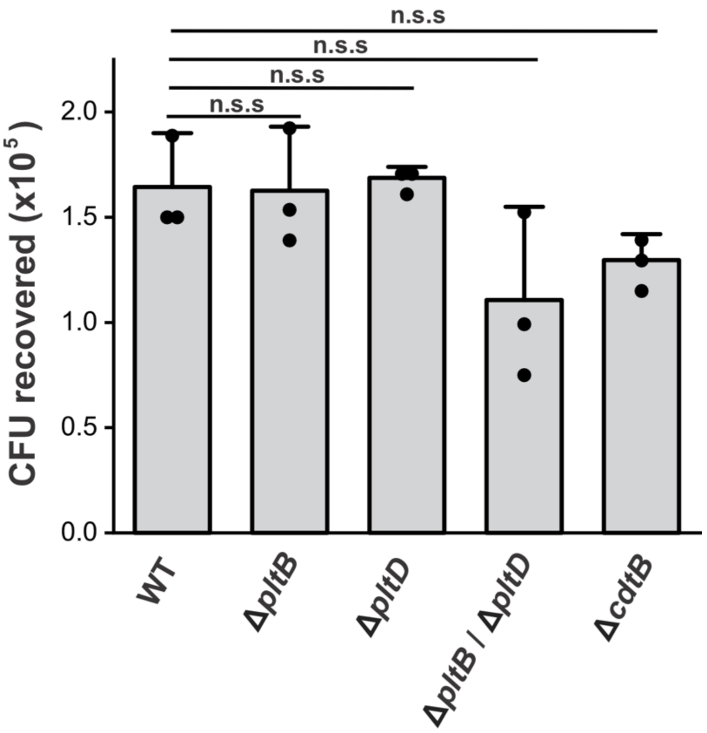

Fig. S7: Recovery of *S. bongori* mutant strains from HeLa cell infections. WT *S. bongori* and the indicated mutant strains were used to infect HeLa cells at a multiplicity of infection (MOI) of 10. Gentamycin was added to the growth medium to prevent the growth of extracellular bacteria. Cells were collected and bacteria were isolated 48 hours post-infection and plated on LB-agar plates to determine the numbers of CFU recovered. statistical significance of the indicated comparisons was determined by Tukey's test: n.s.s., not statistically significant. Error bars represent one standard deviation.

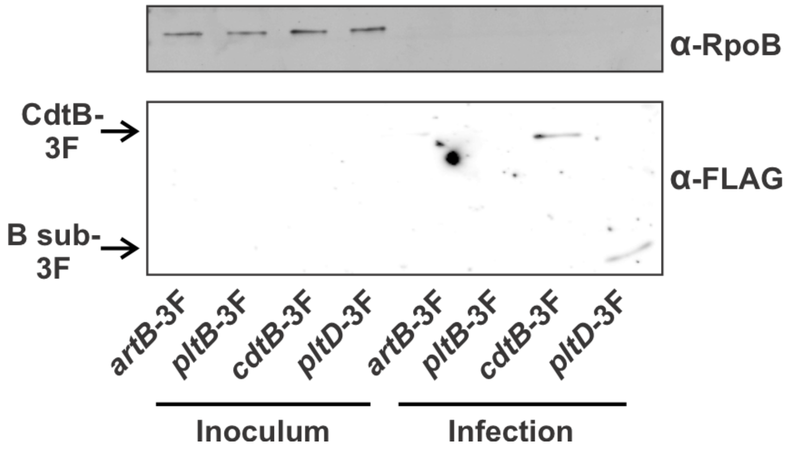

Fig. S8: Replicate experiment comparing the levels of toxin subunits in the infection inoculum to levels 24 hpi. *S. bongori* strains encoding the indicated 3F-tagged genes were used to infect HeLa cells at an MOI of 50. Bacteria were extracted 24 hpi, pelleted, and whole cell lysates were analyzed by western blot using α-FLAG and α-RpoB (loading control) antibodies. Despite higher levels of the inoculum being loaded (RpoB blot), the typhoid toxin subunits PltD and CdtB are only detectable in the infection samples, demonstrating their infection-specific expression pattern. This is a second independent experiment conducted in the same manner as that presented in Fig 5B.
